# Western Pennsylvania Veterinary Medical Association

**Published:** 1891-12

**Authors:** James A. Waugh

**Affiliations:** Secretary


*Western Pennsylvania Veterinary Medical Association.*—The regular monthly meeting was held at Dr. J. C. McNeil's office, Saturday Nov. 14, 1891. Drs. H. S. Jackson, of Sewickly; D. Martin, of McKeesport; and R. Recktenwald, of S. Side, were present as visitors. Minutes of last meeting were read and approved. Jas. A. Waugh, V. S., read a paper on "Electrical Accidents to Domestic Animals," which was discussed by all present, and a vote of thanks was extended to the essayist. Dr. J. C. McNeil volunteered to read a paper at the next meeting. The next meeting will be held on the first Saturday in December.

JAMES A. WAUGH, V. S., *Secretary*.

